# Severe tophaceous gout in the setting of myelofibrosis: A clinical challenge

**DOI:** 10.1016/j.radcr.2025.09.080

**Published:** 2025-10-31

**Authors:** Nicole Chin, Aidan Megally, Omar Zahra, Akshita Gorantla, Shravika Talla, Emad Allam

**Affiliations:** Loyola University Medical Center and Loyola University Chicago, 2160 S First Ave, Maywood, IL 60153, USA

**Keywords:** Gout, Myelofibrosis, Myeloproliferative neoplasm, Uric acid

## Abstract

We illustrate a case of a patient with a history of myelofibrosis, presenting with a clinical picture of presumed infection. Imaging revealed apparent fluid collections and erosions in the ankle/foot, supporting the clinical suspicion of infection. However, operative findings revealed extensive tophaceous gout, likely a consequence of long-standing myelofibrosis. It is important to be aware of this atypical presentation of gout in patients with myelofibrosis.

## Background

Myelofibrosis is a rare chronic blood disorder characterized by progressive fibrosis of the bone marrow, abnormal hematopoiesis, and significant clinical manifestations. It is primarily driven by genetic mutations, most commonly involving the JAK2, CALR, and MPL genes, which result in dysregulated activation of the JAK-STAT signaling pathway [1]. This pathway is integral to cell survival, proliferation, and inflammation and its hyperactivity underpins the pathological features of the disease. The resulting aberrant stem cell activity leads to bone marrow fibrosis, impaired hematopoiesis, and compensatory extramedullary hematopoiesis, contributing to hallmark clinical features such as hepatosplenomegaly, severe anemia, and constitutional symptoms like fatigue, night sweats, and weight loss [2]. Patients with myelofibrosis are also at increased risk for thrombosis and mortality from disease progression and complications [3,4].

Myelofibrosis development can be classified into 2 groups: primary and secondary. Primary arises de novo, whereas secondary myelofibrosis develops as a complication of other myeloproliferative neoplasms (MPNs), such as polycythemia vera or essential thrombocytopenia [2].

While myelofibrosis and MPN patients experience a range of symptoms of varying severity, serum uric acid (SUA) may serve as an important clinical marker [3]. SUA is the final metabolic byproduct of purine nucleotides and is normally predominantly processed in the liver and excreted by the kidneys. Elevated SUA levels, a condition known as hyperuricemia, can indicate increased cell turnover due to the heightened proliferation and turnover of hematopoietic cells seen in MPNs. Impaired renal clearance can also exacerbate hyperuricemia in these patients which can indicate declining renal function or the systemic effects of the disease [5]. Chronic hyperuricemia is associated with an increased risk of developing gout. Gout is an inflammatory arthropathy characterized by the deposition of monosodium urate crystals in soft tissues and joints. In patients with myelofibrosis, hyperuricemia and gout can develop as secondary complications, particularly in the context of advanced disease or therapy-related metabolic disturbances [6]. Clinically, this presents with episodic joint pain, swelling, and inflammation during acute attacks. In chronic or poorly managed cases, this can also lead to the formation of tophi. However, this a complication that is not well documented in the literature [6,7].

## Case presentation

**Fig 1:**
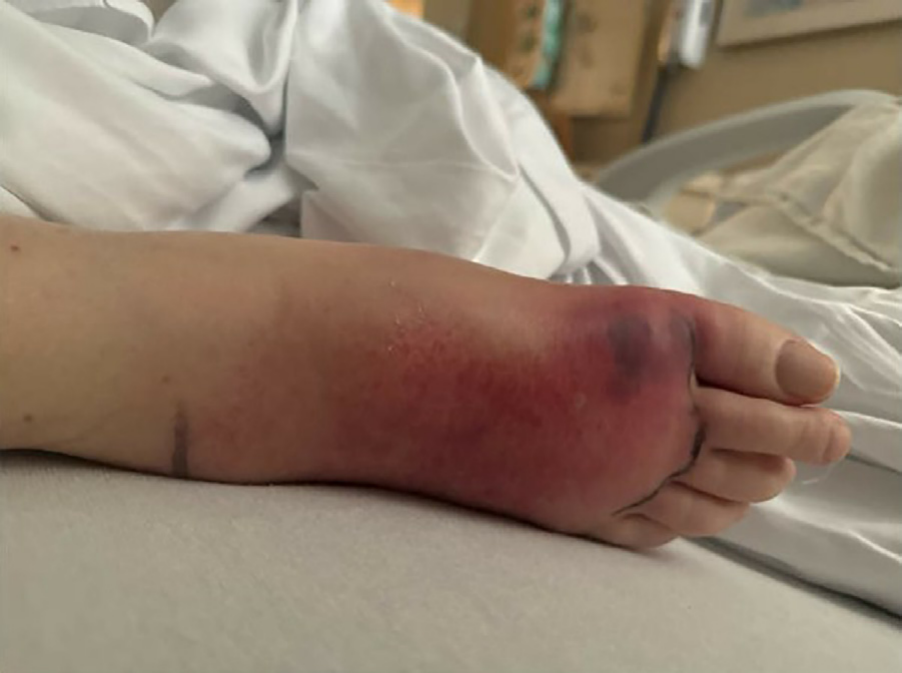
Picture of the right foot shows edema and erythema at the dorsal aspect of the foot, greatest at the forefoot.

**Fig 2:**
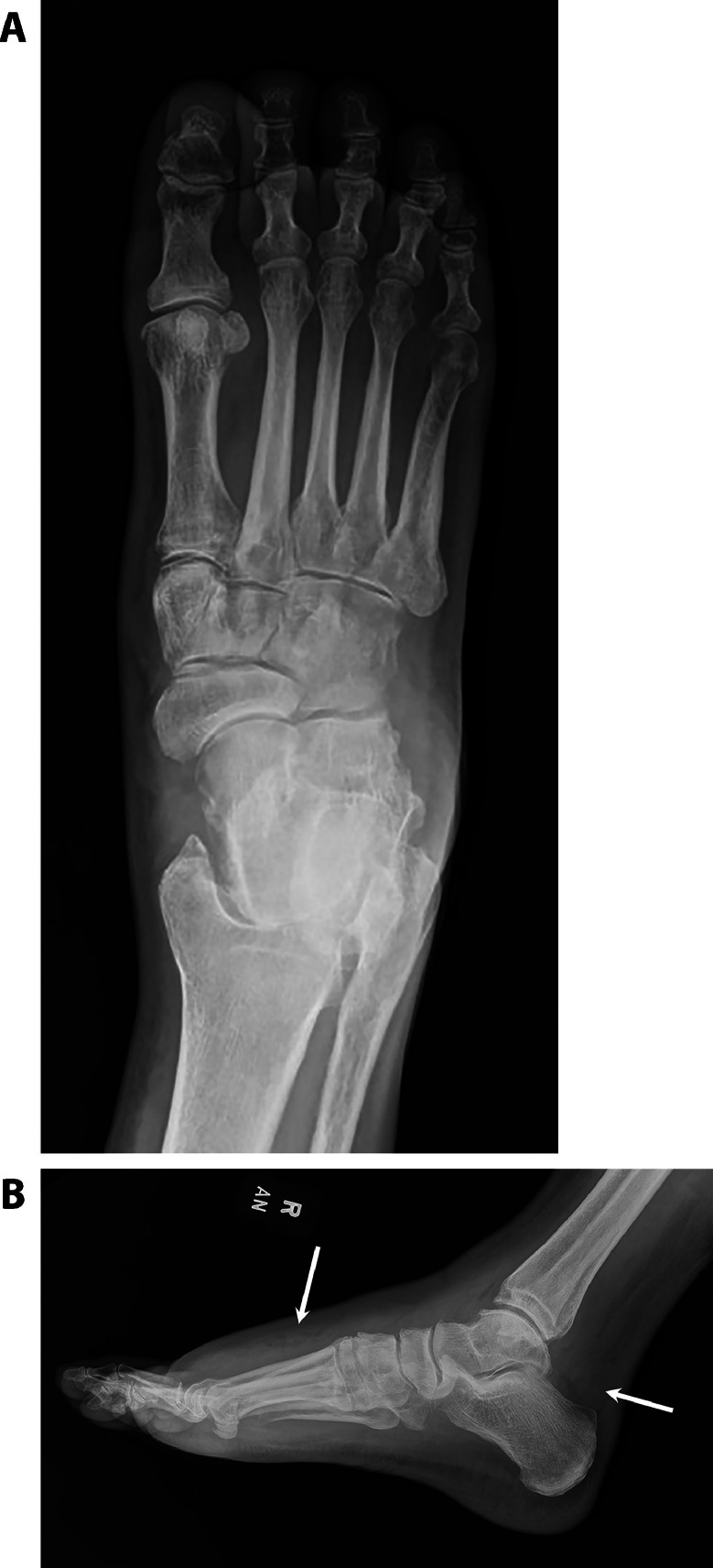
(A) AP radiograph of the right foot demonstrates no acute osseous abnormality. No definite erosions are seen. (B) Lateral radiograph of the right foot demonstrates areas of soft tissue swelling at the dorsal foot and posterior ankle (white arrows).

A 71-year-old female with a history of hypertension, essential thrombocytopenia, and myelofibrosis (JAK2 V617F mutation) on ruxolitinib, azacitidine, and allopurinol presented with epistaxis and right lower extremity swelling and pain, initially concerning for cellulitis (Fig. 1). She was started on ceftriaxone and vancomycin, but after several days without improvement, radiographs were obtained, showing multifocal soft tissue swelling of the right foot without acute osseous abnormalities (Fig. 2). Blood cultures remained negative.

**Fig 3:**
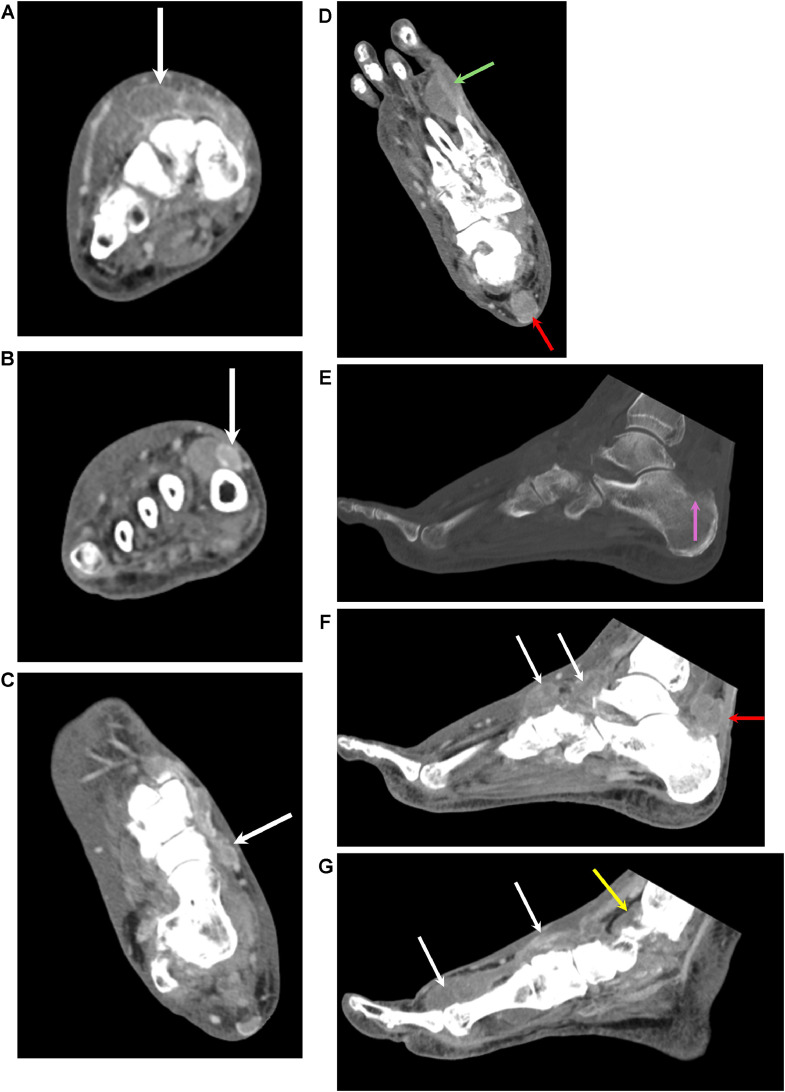
(A and B) Short axis CT of the right foot with IV contrast shows areas of peripherally enhancing soft tissue/fluid collections at the dorsal aspect of the first/second rays (white arrows). There is diffuse subcutaneous edema. (C and D) Long axis CT of the right foot with IV contrast shows areas of peripherally enhancing soft tissue/fluid collections along the first ray (white arrows). This is likely associated with the extensor hallucis longus tendon sheath (green arrow). There is abnormal soft tissue deep to the Achilles tendon (red arrow). There is diffuse subcutaneous edema. (E-G) Sagittal CT of the right foot with IV contrast shows subtle erosion of the posterior superior calcaneus (pink arrow). There are multiple areas of peripherally enhancing soft tissue/fluid collections, including deep to the Achilles tendon (red arrow) and dorsal foot (white arrows). There is an effusion or synovitis in the ankle joint (yellow arrow). There is diffuse subcutaneous edema.

A CT of the right foot was performed the same day as the radiographs and revealed multiple complex fluid collections in the right foot with small bony erosions, raising concern for infection (Fig. 3). Incision and drainage of the right foot was performed 2 days after the CT. Intraoperatively, significant tophaceous material and viscous white drainage were noted, consistent with gout. Cultures from the drainage, including aerobic, anaerobic, fungal, and acid-fast bacilli cultures were negative. In retrospect, gout was a more appropriate diagnosis than infection, given negative blood cultures and lack of response to antibiotics. The patient also had a history of elevated uric acid and her uric acid on admission was 8.2 mg/dL (reference range 2.5 to 6.5 mg/dL in women).

**Fig 4:**
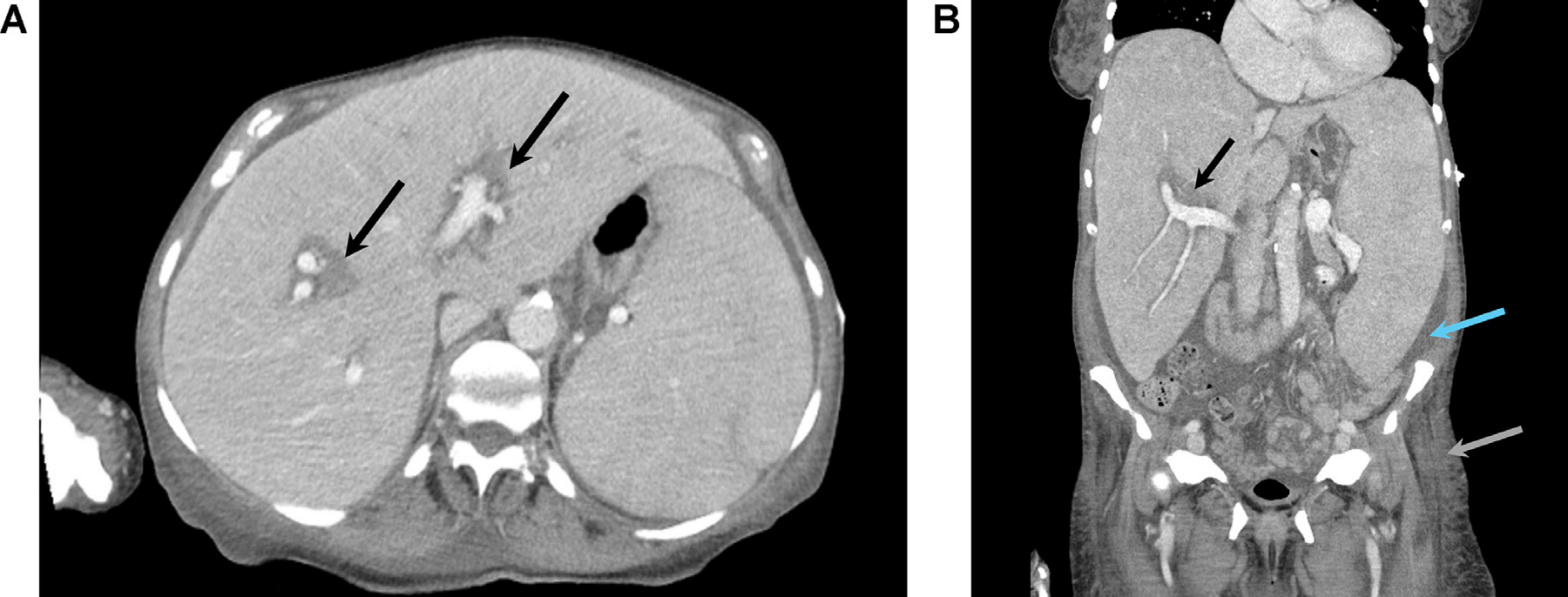
(A) Axial CT of the abdomen with IV contrast shows hepatosplenomegaly. There is periportal low attenuation, likely representing periportal edema (black arrows). (B) Coronal CT of the abdomen with IV contrast shows hepatosplenomegaly. There is periportal edema (black arrow), ascites (blue arrow), and anasarca (gray arrow).

Treatment options were limited due to acute kidney injury, cytopenia, and immunosuppression, precluding NSAIDs, colchicine, corticosteroids, or IL-1 inhibitors. Her kidney function rapidly declined and her mental status acutely worsened. CT of the chest, abdomen and pelvis was performed 3 days after the foot surgery and showed hepatosplenomegaly, consistent with myelofibrosis (Fig. 4). No source of infection was identified. Her pressor requirements increased and the patient’s family elected to pursue comfort care. The patient died 3 days after the CT of the chest, abdomen and pelvis.

## Discussion

This case report exemplifies the complexity of myelofibrosis and the atypical clinical manifestation of gout. Gout arising in the setting of secondary myelofibrosis is scarcely reported in the literature, making this presentation particularly unique [6,7]. We hope to highlight gout as a complication of myelofibrosis, even in patients with several years of managed secondary myelofibrosis, and to discuss the imaging appearance of the disease.

A distinguishing feature in this case was the rapid development of tophaceous gout, with significant tophi formation and joint involvement. This contrasts with chronic tophaceous gout, where tophi typically develop only after prolonged hyperuricemia or repeated acute gout flares [8]. Patients with MPNs, including secondary myelofibrosis, may have accelerated deposition of urate crystals due to the high cellular turnover and metabolic disturbances inherent to these disorders.

Although some studies indicate that SUA levels may not differ significantly between patients with secondary myelofibrosis and controls, our case underscores the impact of sustained moderate hyperuricemia in the pathogenesis of gout [3]. Even marginal elevations in SUA, combined with factors like cytopenia and immunosuppression, can predispose these patients to severe and atypical gout presentations. Elevated SUA has been associated with increased mortality in patients with myelofibrosis [3].

Clinicians and radiologists must remain vigilant for the possibility of tophaceous gout when evaluating soft tissue swelling, particularly in patients with myeloproliferative disorders. Imaging findings such as bony erosions and complex fluid collections can mimic infectious processes, further complicating diagnosis in these cases. Early consideration of gout in the differential diagnosis could prevent unnecessary antibiotic exposure and guide more targeted management strategies.

## Conclusion

Extensive tophaceous gout may be seen in MPNs including myelofibrosis. The imaging appearance can be very similar to infection, but it is important to consider gout in the differential diagnosis in such patients due to the treatment implications. Multidisciplinary collaboration and knowledge of the history of myelofibrosis are important to arrive at the correct diagnosis.

## Patient consent

Informed consent for this case was obtained from the patient’s next of kin.
